# Acupuncture as Adjuvant Therapy for Glaucoma: Protocol for a Randomized Controlled Trial

**DOI:** 10.2196/57888

**Published:** 2024-10-08

**Authors:** Yi-Fang Liao, Yu-Chen Lee, Hui-Ju Lin, Yi-Ching Shao

**Affiliations:** 1 Department of Chinese Medicine China Medical University Hospital Taichung Taiwan; 2 Graduate Institute of Acupuncture Science College of Chinese Medicine China Medical University Taichung Taiwan; 3 Department of Ophthalmology Eye Center China Medical University Hospital Taichung Taiwan; 4 School of Chinese Medicine China Medical University Taichung Taiwan; 5 Graduate Institute of Biomedical Sciences College of Medicine China Medical University Taichung Taiwan

**Keywords:** acupuncture, open-angle glaucoma, optical coherence tomography, intraocular pressure, glaucoma, adjuvant therapy, optic neuropathy, disease progression, ophthalmic disorders, optic, conventional treatment, efficacy, adjunctive therapy

## Abstract

**Background:**

Glaucoma is a chronic progressive optic neuropathy that necessitates lifelong treatment to reduce the decline of the optic nerve. Due to the extended and continuous treatments required for patients, complementary therapies are often considered alongside conventional treatments to enhance the effectiveness of the treatment. Acupuncture has demonstrated the potential to lower intraocular pressure in previous clinical trials, making it a promising glaucoma intervention.

**Objective:**

The primary objective of this study is to conduct a single-center randomized control trial involving patients with glaucoma. Acupuncture will be evaluated as an adjunctive therapy. The trial aims to explore its effectiveness for glaucoma.

**Methods:**

In this single-center randomized controlled trial, participants (N=50) with primary open-angle glaucoma will be randomly assigned to the treatment group, receiving ophthalmic acupuncture with “De Qi” sensation, or the control group, receiving minimum acupuncture stimulation on nonophthalmic acupoints. The intervention will consist of weekly acupuncture treatments for a total of 6 sessions. Participants will be assessed at 8 time points, which are baseline, during the intervention (6 times), and at a 3-month follow-up. The primary outcome measure is a change in the intraocular pressure before and after each acupuncture treatment. Secondary outcomes will include measurements of heart rate and blood pressure before and after acupuncture, best-corrected visual acuity, visual field, optical coherence tomography, optical coherence tomography angiography, the Glaucoma Symptom Scale, and the Glaucoma Quality of Life-15 questionnaire.

**Results:**

Recruitment of participants for the trial commenced on June 28, 2023. A total of 10 participants have been enrolled to test the feasibility of the experiment. We anticipate that the preliminary data from this trial will be completed by December 2025.

**Conclusions:**

This trial uses rigorous methodology and comprehensive outcome measurements to assess the clinical efficacy of acupuncture as an adjunctive therapy for glaucoma, providing valuable insights for future clinical treatment guidelines.

**Trial Registration:**

ClinicalTrials.gov NCT05753137; https://clinicaltrials.gov/study/NCT05753137

**International Registered Report Identifier (IRRID):**

DERR1-10.2196/57888

## Introduction

Glaucoma is a chronic optic nerve disease involving progressive loss of retinal ganglion cells, necessitating long-term treatment with minimal side effects. Some patients may choose to supplement their conventional medical interventions with complementary or alternative medicine [1-3].

In complementary and alternative therapies, there is literature on treating glaucoma with acupuncture, auricular acupressure [4], herbal medicine [5,6], nutritional supplements [7], and Ayurvedic medicine [8]. Possible mechanisms for acupuncture treatment have been proposed, including the release of neurochemical substances through acupuncture stimulation; the influence of acupuncture on ocular blood flow parameters [9,10]; and the upregulation of neurotrophic factors, such as nerve growth factor and brain-derived neurotrophic factor expression in the retina [11]. In an observation study on acupuncture treatment for glaucoma, Law et al [12] conducted a prospective study including 11 patients with glaucoma and observed a short-term increase in intraocular pressure (IOP) after acupuncture [12]. However, another study presented a different perspective, suggesting that acupuncture at the acupoints Jingming (BL1) and Qiuhou (EX-HN7) was beneficial in reducing IOP [13]. Systematic literature reviews have also supported the potential of acupuncture in lowering IOP and improving visual fields [14].

The objective of this single-center randomized control trial is to evaluate the clinical efficacy of acupuncture in the context of glaucoma and provide a reference for future clinical treatment guidelines.

## Methods

### Design and Setting

The study is designed as a single-center, parallel-arm, randomized controlled trial. It will take place in the clinic of China Medical University Hospital. The trial will be conducted from June 2023 until 50 patients finish acupuncture treatment and follow-up assessment. This protocol is reported following the SPIRIT (Standard Protocol Items: Recommendations for Intervention Trials) 2013 statement, which defines standard protocol items for clinical trials [15].

The main purpose of the trial is to investigate the effectiveness of acupuncture in the treatment of glaucoma, including its potential to decrease IOP and reduce optic nerve damage. All participants will be randomly assigned to 2 groups: the treatment group and the control group. Participants in the treatment group will receive acupuncture treatment targeting acupuncture points related to ophthalmology, while participants in the control group will receive minimum acupuncture targeting nonophthalmic acupuncture points.

**Figure 1:**
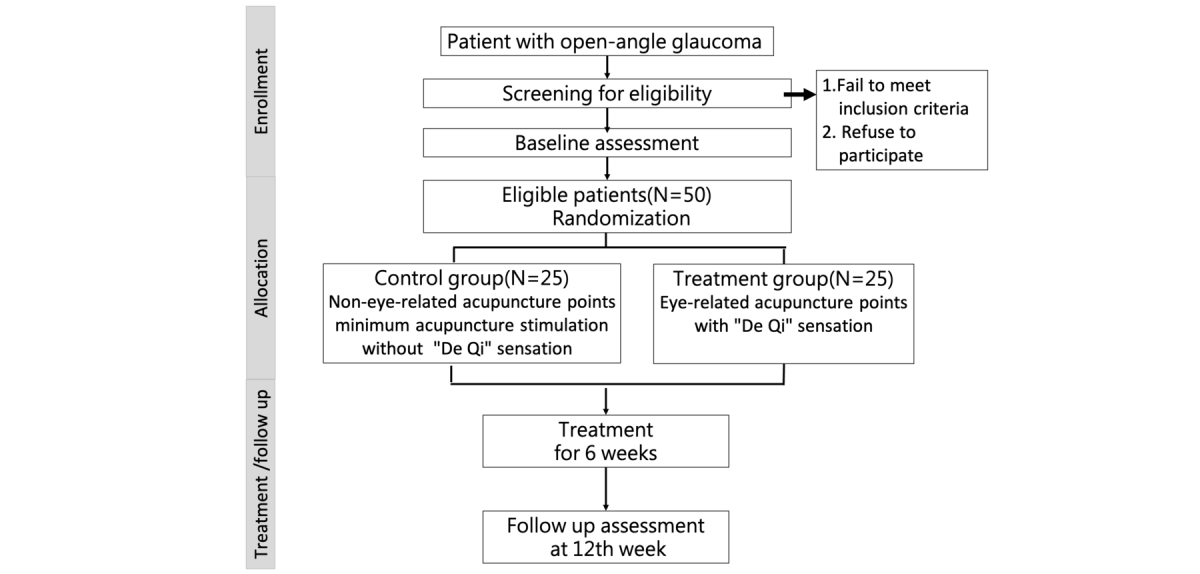
The enrollment process of the trial.

In addition, all participants will undergo ophthalmic examinations and follow-up. Figure 1 illustrates the enrollment process of the trial. The assessments will include measurements of IOP, heart rate (HR), blood pressure (BP), best-corrected visual acuity (BCVA), central corneal thickness, visual field (VF), optical coherence tomography (OCT) imaging, optical coherence tomography angiography (OCTA), and visual function questionnaire.

### Sample Size Calculation and Power

Assuming an effect size of 0.40 and a type I error (α) of .05, a sample size of 40 people (20 in each group) is required to achieve a statistical power of 95% CI. Assuming a dropout rate of 25%, a total of 50 people (25 in each group) is needed (Textbox 1).

**Inclusion and exclusion criteria.**

**Inclusion criteria**

- Primary open-angle glaucoma was diagnosed at least 3 months ago
- Diagnosed with mild or moderate open-angle glaucoma
- Use 1 or 2 kinds of glaucoma drugs
- Age ≥20 years old
- All participants will be required to sign an informed consent form and cooperate with the experimental procedures

**Exclusion criteria**

- Accept any ophthalmic laser or surgery within 1 year
- High myopia (≤6.0D)
- Use of any drugs that affect intraocular pressure
- Visual acuity with correction lower than 0.2
- Previous or existing uveitis or retinopathy
- Unable to receive acupuncture treatment continuously or allergic to acupuncture needles
- Pregnancy or breastfeeding
- Refusal to sign the informed consent form

### Ethical Considerations

Ethics approval was obtained from the institutional review board of China Medical University Hospital (CMUH111-REC3-210), and the trial was registered on ClinicalTrials.gov (NCT05753137). The informed consent discussion will take place before the trial. The informed consent form will be explained in clear and understandable language by a hospital doctor, and it will cover the intervention, risks, benefits, and patient rights in the trial. The informed consent form will be signed by the patients. If a patient wishes to withdraw from the trial, we will use the available data for the final analysis and accommodate their wishes accordingly. Confidentiality of information will be assured to the participants. All data will be anonymized or deidentified. As indicated on the recruitment poster, participants received NT $1000 (US $31) as compensation.

### Randomization and Allocation Concealment

The participants will be randomly assigned to 2 groups with a 1:1 ratio using random number generation in Microsoft Excel. A computer-based simple random sampling method without stratification with a 1:1 ratio will be used. After the random allocation is completed, the researchers will assign sequential numbers (1-50) to the participants and prepare 50 folders. The appearance of the folders will be identical for both groups, and the section containing the participant’s group and the intervention measures will be sealed. When participants complete the informed consent process, the acupuncturist will unseal the section and begin the acupuncture treatment.

### Blinding

Upon revealing the treatment allocation, the acupuncturist will proceed to administer acupuncture based on the patient’s assigned group. Throughout the entire experiment, all sterile needles will have the same appearance, thickness, and size. Only the acupuncturist will be aware of the patient’s group allocation.

Participants will be informed that there are 2 different acupuncture groups, but they will only receive 1 of them. In addition, they will undergo treatment in separate rooms to prevent any communication between them. At the end of the trial, participants will be orally questioned about whether they are aware of the treatment strategy they received, confirming the effectiveness of the blinding implementation. Throughout the entire study, there will be no communication between the acupuncturist and the participants, as well as between the acupuncturist and the assessors. This is to maintain the blinding of the participants, researchers, assessors, and statisticians to the group allocation.

### Interventions

Patients who meet the inclusion criteria at the Ophthalmology Clinic of China Medical University Hospital in Taiwan will be enrolled in a trial and randomly assigned to different groups. Both groups of patients will receive ophthalmic prescriptions and care. Acupuncture treatment will be performed using sterile acupuncture needles manufactured by “Jia Shun” (FDA 000470).

The acupuncturist will disinfect the acupoints using alcohol pads containing 70% alcohol. Then, they will apply acupuncture treatment by inserting the needles perpendicularly into the muscle layer, followed by up and down manipulation to induce the “De Qi” sensation in the treatment group. However, the control group will not experience the “De Qi” sensation; the needles will penetrate the skin, but the needling process will be halted as soon as the patients start feeling stimulation. During the 20-minute acupuncture session, the patients will maintain either a supine position or a sitting position. The acupuncturist is not allowed to communicate with the patients during the trial, except to inquire about the sensation of “De Qi.” In addition, after patients are enrolled in the trial, if no serious adverse events occur, the ophthalmologist evaluator will remain blinded and isolated until the completion of the trial at the 12th week to maintain the integrity of blinding in the trial.

### Treatment Group

**Figure 2:**
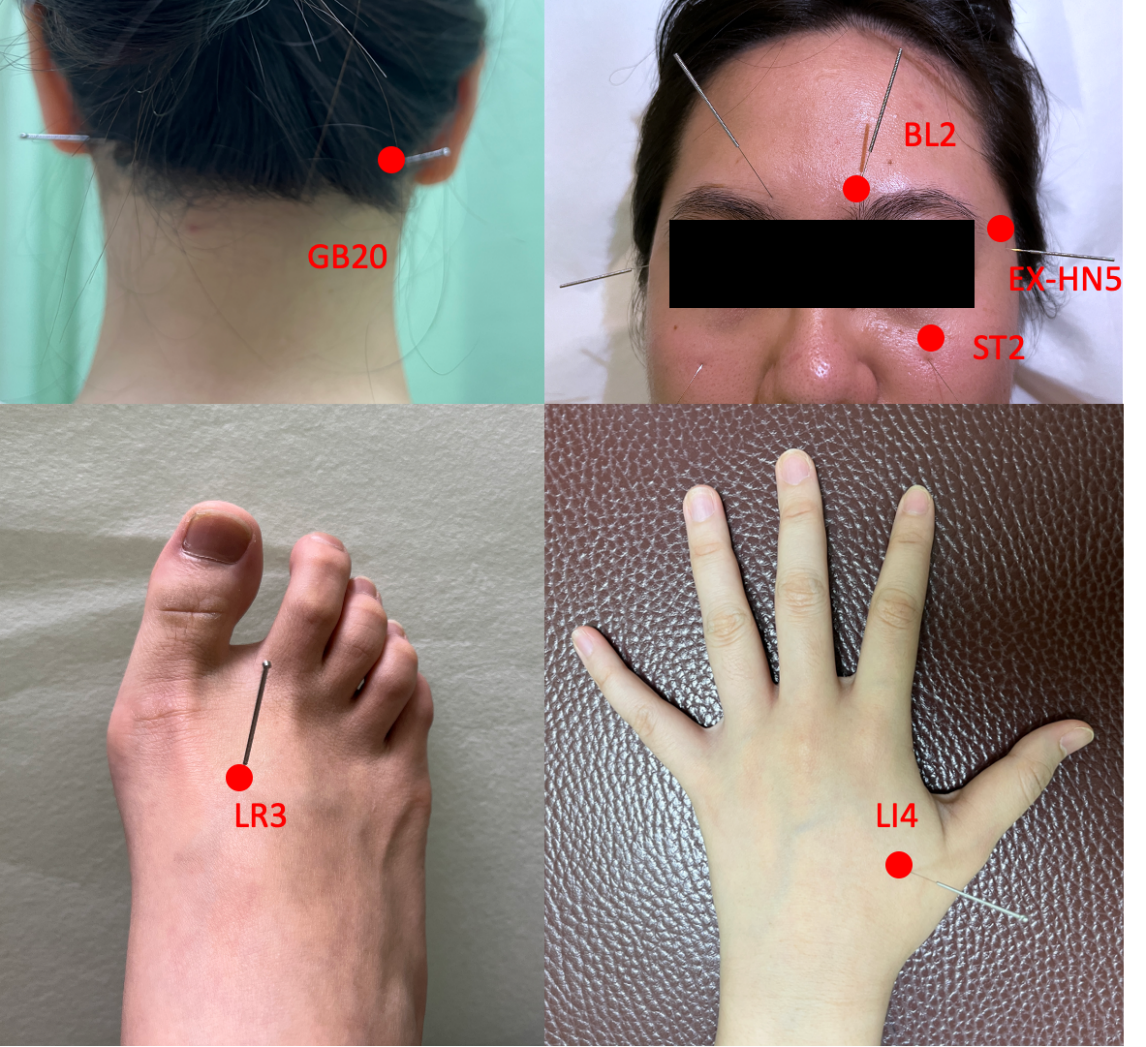
Acupoints used in the treatment group: Fengchi (GB20), Cuanzhu (BL2), Sibai (ST2), Taiyang (EX-HN5), Hegu (LI4), and Taichong (LR3).

In addition to routine ophthalmic prescription treatments, patients in the treatment group will receive traditional Chinese medicine–style acupuncture at the following bilateral acupoints once a week for 6 weeks: Fengchi (GB20), Cuanzhu (BL2), Sibai (ST2), Taiyang (EX-HN5), Hegu (LI4), and Taichong (LR3), for a total of 6 acupoints. The acupoint selection in this group is specific to treat symptoms of glaucoma. Figure 2 shows acupoints used in the treatment group.

### Control Group

**Figure 3:**
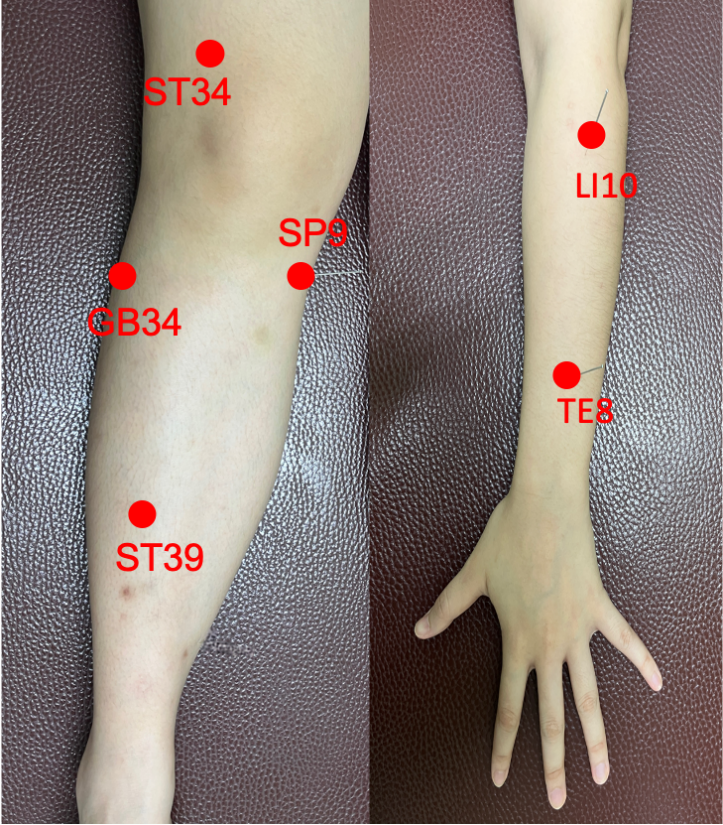
Acupoints used in the control group: Yinlingquan (SP9), Liangqiu (ST34), Xiajuxu (ST39), Yanglingquan (GB34), Shousanli (LI10), and Sanyangluo (TE8).

In addition to routine ophthalmic prescription treatments, patients in the control group will receive minimum acupuncture stimulation at the following bilateral acupoints once a week for 6 weeks: Yinlingquan (SP9), Liangqiu (ST34), Xiajuxu (ST39), Yanglingquan (GB34), Shousanli (LI10), Sanyangluo (TE8), for a total of 6 acupoints. Figure 3 shows acupoints used in the control group. The nonophthalmological acupoints are not indicated for the treatment of ophthalmological pathologies and are not reported to improve ophthalmological function [16].

### Outcome Measures

The primary outcome is the change in IOP as measured using the CT-80 Computerized Tonometer (TOPCON). Before the start of the trial, the participants’ baseline IOP will be measured. Subsequently, IOP data will be collected before each acupuncture treatment and 15 minutes after the treatment. By comparing these measurements, the effect of acupuncture treatment on IOP can be assessed.

The secondary outcomes include the long-term effects of acupuncture on the eyes. Baseline measurements and measurements after the completion of the sixth acupuncture session will be taken using OCTA (AngioVue, QuickVue version, Optovue Inc) to assess the vessel density of the radial peripapillary capillary and macula. In addition, VF, OCT, BCVA, central corneal thickness, and 2 questionnaires will be recorded at baseline and during follow-up at week 12. VF measurements will be conducted using the Humphrey Field Analyzer 3 (ZEISS) to measure the mean deviation, pattern SD, and VF index values. OCT imaging will be performed using the ZEISS HD-OCT model 5000, and measurements of the retinal nerve fiber layer, ganglion cell complex and symmetry, and the cup-to-disc ratio will be recorded. The 2 questionnaires used in the trial are the Glaucoma Symptom Scale (GSS; score range 0-100) and the Glaucoma Quality of Life-15 (GQL-15; score range 0-75) [17,18]. These questionnaires are used to quantify the extent of functional impairment or inconvenience in patients with glaucoma. In addition, to ensure the safety of participants during the trial and to address other relevant considerations related to the pathophysiology of glaucoma, BP and HR measurements will be taken at baseline, before and after acupuncture sessions, and during follow-up assessments. Table 1 depicts the trial schedule, illustrating the journey of patients from initiation to the completion of the trial.

**Table 1:** Trial schedule, showing the journey of patients from initiation to the completion of the trial.

Time point		Enrollment	Treatment		Follow-up
		Visit day 1	Week 1-6	Week 6 (after acupuncture)	Week 12
**Eligibility screen**		✓			
**Informed consent**		✓			
**Allocation**		✓			
**Acupuncture**					
	Treatment group		✓		
	Control group		✓		
**Data collection**					
	Intraocular pressure	✓	✓	✓	✓
	Blood pressure	✓	✓	✓	
	Heart rate	✓	✓	✓	
	OCTA^a^	✓		✓	
	OCT^b^	✓			✓
	BCVA^c^	✓			✓
	Visual field	✓			✓
	Central corneal thickness	✓			✓
	GSS^d^	✓			✓
	GQL-15^e^	✓			✓

^a^OCTA: optical coherence tomography angiography.

^b^OCT: optical coherence tomography.

^c^BCVA: best-corrected visual acuity.

^d^GSS: Glaucoma Symptom Scale.

^e^GQL-15: Glaucoma Quality of Life-15.

### Adverse Events

The study will document adverse reactions including the location of adverse reactions, the acupuncture site, the time of occurrence, the duration of symptoms, and the recovery status. In addition, appropriate medical resources and financial assistance, such as arranging emergency or outpatient care, will be provided to participants as needed.

### Statistical Analysis

The statistical analysis will be performed by a statistician from the YCL’s laboratory who is completely blind to patient allocation, using SPSS software (version 29.0; IBM Corp). The demographic data between the 2 groups, including sex, age, initial IOP, HR and BP, and BCVA will be compared using the 1-tailed Student *t* test. For the data from the 2 glaucoma questionnaires, a 1-way ANOVA will be used. In addition, a repeated measures analysis will be conducted in the trial. The data will be presented as mean (SD), and a *P* value of less than .05 will be considered statistically significant. Subgroup analysis will be conducted based on factors, such as age; gender; changes in IOP before and after treatment; VF, OCT, and OCTA data; and central corneal thickness, among others. In cases of small sample size, the Mann-Whitney *U* test and the Wilcoxon signed-rank test will be used if data do not follow normal distribution. Missing data will be analyzed using pairwise deletion.

## Results

Recruitment of participants for the trial commenced on June 28, 2023. A total of 10 participants have been enrolled to test the feasibility of the experiment. We anticipate that the preliminary data from this trial will be completed by 2025.

## Discussion

### Expected Findings

Glaucoma, the second leading cause of blindness globally, has an alternative medicine usage rate of approximately 5% to 14% [3,19]. Our study delves into the potential of acupuncture as an adjunctive therapy for glaucoma, drawing inspiration from compelling evidence from previous research. The selection of acupuncture points was based on the following arguments. Takayama et al [20] conducted acupuncture on 11 patients with open-angle glaucoma, targeting the acupoints BL2, M-HN9, ST2, ST36, SP6, KI3, LR3, GB20, BL18, and BL23. This treatment showed a significant reduction in IOP, had effects on the short posterior ciliary artery, and improved retrobulbar circulation [9]. The ophthalmic artery is responsible for the main blood supply to the eyes, while the anterior cerebral artery, middle cerebral artery, and ophthalmic artery are downstream branches of the internal carotid artery. In a series of studies, it has been observed that the acupoints GV20, GB20, and GB34 are associated with increased blood flow velocity in the anterior cerebral artery and middle cerebral artery, as well as increased cerebral blood flow [21-23]. In a mouse model of traumatic optic neuropathy, the acupoint GB20 significantly altered the expression of posttraumatic retinal genes. This suggests that acupuncture may target specific genes involved in the treatment of glaucoma, such as MT3 (associated with ocular neovascularization) and FBN1 (related to macular degeneration) [24].

LR3 is an acupoint located on the liver meridian. It is known for its functions related to “soothing the liver,” “regulating blood,” and “affecting the eyes.” According to traditional Chinese medicine theory, LI4 and LR3 are considered intermediate stations for the gathering and transformation of energy within the body. The combination of acupuncture stimulation at these 2 acupoints is referred to as “the 4 gates.” It is commonly used to promote the circulation of Qi and blood throughout the body and can influence brain areas involved in BP regulation, thus having a regulatory effect on BP [25,26]. LR3 activation leads to the activation of the visual cortex BA 19 and the inhibition of the ipsilateral BA 17 and 18 [27,28]. In addition, LR3 also results in a significant reduction in vascular resistance in the central retinal artery and short posterior ciliary artery [20].

Patients with migraine have an increased risk of developing open-angle glaucoma, and the most commonly used acupoints in acupuncture for this condition are EX-HN5 and GB20 [29-31]. LI4 is also believed to be associated with pain relief, particularly in the treatment of muscle or nerve pain or paralysis in the head and face, and has shown significant benefits [32]. Based on these beneficial effects, we chose the acupoint combination of EX-HN5, GB20, BL2, ST2, LI4, and LR3 for treatment.

The effectiveness of glaucoma treatment extends beyond clinical measures and should consider the holistic well-being of individuals. Therefore, this study assessed the influence of acupuncture on the daily life convenience and functionality of individuals with glaucoma, using the GSS and GQL-15 questionnaires. This includes evaluating the psychological, social, and functional dimensions that contribute to their overall well-being. By extending the focus to these elements, we gain a better understanding of the treatment’s effects on the individuals’ day-to-day experiences and functionality. In this trial, the impact of acupuncture on the daily life convenience and functionality of patients with glaucoma was quantified using the GSS and GQL-15 questionnaires. The GSS is a simple questionnaire used to measure symptoms and functional loss in daily life. In the 1998 version of the GSS by Lee et al [17], the questionnaire consists of 10 items, and each eye is rated on a 5-point scale ranging from 0 (very bothersome) to 4 (not bothersome). The scores are then converted to a scale of 0 to 100 for statistical analysis, where 0 represents the presence of significant problems and 100 indicates the absence of problems [18]. In addition, the GQL-15 by Nelson et al[18] assesses the impact of glaucoma on the quality of life of patients. It consists of 15 items related to various aspects of daily life, such as reading newspapers and walking after dark. Each question is rated on a scale of 0-5. The total score ranges from 0 to 75, with a higher score indicating a poorer quality of life.

### Conclusions

This study strengthens our understanding of acupuncture as a potential adjunctive therapy for glaucoma, The methodological rigor, including a randomized controlled trial, enhances the credibility of findings. The comprehensive evaluation, incorporating diverse outcome measures such as IOP, cardiovascular parameters, and various ophthalmic assessments, provides valuable insights into acupuncture’s impact on glaucoma management. The 12-week observation period contributes valuable information on both short-term and potential long-term effects.

However, limitations include the single-center design, affecting generalizability, and a modest sample size that may compromise statistical power. The study’s duration may not fully capture long-term effects.

## Appendix

Multimedia Appendix 1 CONSORT-eHEALTH (V 1.6.1).

## Abbreviations

BCVA: best-corrected visual acuity
BP: blood pressure
GQL-15: Glaucoma Quality of Life-15
GSS: Glaucoma Symptom Scale
HR: heart rate
IOP: intraocular pressure
OCT: optical coherence tomography
OCTA: optical coherence tomography angiography
SPIRIT: Standard Protocol Items: Recommendations for Intervention Trials
VF: visual field

## Data Availability

Data sharing does not apply to this article as no datasets were generated or analyzed during this study. For any additional inquiries, please contact the corresponding author directly.
